# Does optimized adherence support improve treatment outcomes in RR / MDR-TB patients on 18–20 months regimen in Tbilisi, Georgia?

**DOI:** 10.3855/jidc.13783

**Published:** 2021-09-29

**Authors:** Tinatin Jomidava, Mohammed Khogali, Yuliia Sereda, Zaza Avaliani, Malkhaz Davitashvili, Mikheil Madzgharashvili, Nestan Tukvadze, Lali Chaphurishvili, Mamuka Chincharauli, Maia Kipiani

**Affiliations:** 1 National Center for Tuberculosis and Lung Diseases, Tbilisi, Georgia; 2 UNICEF / UNDP / World Bank / WHO Special Programme for Research and Training in Tropical Diseases (TDR), World Health Organization, Geneva, Switzerland; 3 Joint Tuberculosis, HIV and Viral Hepatitis Program, Division of Health Emergencies and Communicable Diseases, WHO Regional Office for Europe, Copenhagen, Denmark; 4 The University of Georgia, Tbilisi, Georgia

**Keywords:** Drug-resistant tuberculosis, adherence, treatment outcomes, before-and-after study, SORT IT

## Abstract

**Introduction:**

Adherence to second-line antituberculosis drug is challenging. A combination of strategies needs to be implemented to achieve adherence. In Georgia an optimized adherence support (OAS) – a package of education, psychosocial support and adherence counselling – was added to the already existing package of adherence support (supervised treatment, adherence incentives, transport cost reimbursement) to improve adherence and increase treatment success. We assessed the additive benefits of OAS on adherence and treatment outcomes.

**Methodology:**

This was a before and after cohort study using routine programme data in the National Center for Tuberculosis and Lung Diseases in Tbilisi. All adult rifampicin- and multidrug-resistant tuberculosis (RR/MDR-TB) patients enrolled for treatment under directly observed therapy in the NCTLD during the period before (June 2015 – January 2016) and after (June 2017 – January 2018) were included in the study. Primary outcomes were: i) adequate adherence defined as ≥ 85% of days covered by TB medication during the whole treatment period; ii) final treatment outcomes.

**Results:**

Of 221 RR/MDR-TB, most patients were male (76%, N = 167) with a mean age of 41 ± 14 years. Adherence data was available for 111 patients in the ‘before’ and 97 patients in the ‘after’ cohort. Adequate adherence was achieved by 62% (69/111) in the ‘before’ and 70% (68/97) in the ‘after’ cohort (*p* = 0.290). Overall treatment success was 64% (73/114) and 63% (67/107) in the ‘before’ and ‘after’ cohorts respectively (*p* = 0.937).

**Conclusions:**

Implementation of OAS had modest effect on adherence and had no additive benefits on treatment outcomes among RR/MDR-TB patients on 18–20 months regimen.

## Introduction

Rifampicin-resistant (RR) and multidrug-resistant (MDR) tuberculosis (TB) is an infection caused by mycobacteria that do not respond to rifampicin (RR-TB) or both rifampicin and isoniazid (MDR-TB), the two most powerful first-line antituberculosis drugs [1]. Compared to drug-sensitive TB, drug-resistant TB has limited treatment options, longer duration and higher costs of treatment, and more drug toxicity [2]. These factors adversely impact the physical and mental well-being of patients [3]. Rates of treatment noncompletion and interruption in RR/MDR-TB patients are thus significantly higher than in drug-susceptible TB patients [4]. Globally, only 56% of all the RR/MDR-TB cases were on treatment [5]. Of those who initiated treatment in 2016, about 15% were lost to follow-up (LTFU) from treatment, defined as interrupting treatment for more than two months, and only half were successfully treated [5]. Treatment non-adherence and treatment interruptions diminish the quality-of-life of people living with RR/MDR-TB and increase transmission of drug-resistant organisms in the community [6]. Several factors are associated with non-adherence, such as financial, social, personal and psychological barriers [2,3]. Therefore, several strategies need to be combined, adapted and used to achieve adherence [7]. The World Health Organization (WHO) recommends a combination of interventions that include a patient-centered package and a supervision option according to patients needs [8]. These interventions can be summarized into four groups: a) supervision of treatment intake through directly observed therapy (DOT) or video-observed therapy (VOT); b) reminder systems (phone calls, short message services); c) education and psychosocial support; and d) nutrition and financial support [8,9]. In Georgia, the prevalence of RR/MDR-TB is 12% among new cases and 31% among previously treated cases, which is above the global average [5]. As in other settings, non-adherence to second-line antituberculosis treatment poses a great challenge to TB control efforts. Since 2003, the National Center for Tuberculosis and Lung Diseases (NCTLD) in Georgia has been implementing different strategies to enhance adherence. These include DOT and VOT, adherence incentives and financial support to cover transport costs for patients. Despite these strategies, treatment success among RR/MDR-TB patients is 65%, which is far below the End TB Strategy target of 90% [5]. In 2017, optimized adherence support (OAS) was introduced to address the gap in education and psychosocial support services. The OAS is a patient-centered package of services delivered by trained consultants who collaborate with clinicians and provide education, psychosocial support, adherence counseling for patients and investigate cases of non-adherence throughout the period of treatment. The effectiveness of OAS in addition to other strategies has not yet been evaluated. Several systematic reviews have evaluated a broad range of interventions to improve adherence to treatment and have concluded that a patient-centered package of interventions is more likely to improve TB outcomes compared with supervision or financial support alone [4,7,9,10]. Other studies have shown that educational, social and psychological support is effective in improving adherence and decreasing rates of LTFU [11,12]. However, the magnitude of the effect of these interventions varies depending on the implementation modalities and settings [4]. A PubMed search revealed no studies that have assessed the effect of a comprehensive patient-centered package on RR/MDR-TB treatment outcomes in Georgia or in the European region. The findings of this study will inform the NCTLD in Georgia and other TB programs in the region whether introduction of OAS in addition to supervision and financial support improves adherence to treatment and leads to better cure rates. This information is important to guide planning and allocation of resources. In this study, we aimed to assess the effectiveness of adding OAS to the standard package of services (supervision and financial support) among all adult RR/MDR-TB patients enrolled for TB treatment for the period before (between June 2015 and January 2016, ‘before’ cohort) and after (between June 2017 and January 2018, ‘after’ cohort) the implementation of OAS in Tbilisi, Georgia. Specific objectives were to compare the: i) socio-demographic and clinical characteristics; ii) adherence to treatment; iii) final treatment outcomes.

## Methodology

### Study Design

This was a before and after cohort study using routine programme data.

### Settings

Georgia is a country in Eastern Europe with a population size of about 3.7 million. The capital and largest city is Tbilisi with a population of about 1.5 million people.

### Study site

The study was conducted in the NCTLD, Tbilisi. The NCLTD has been offering free of charge TB care and treatment services for all TB patients in Tbilisi since 2001.

### Diagnosis and treatment of RR/MDR-TB

Diagnosis and treatment of RR/MDR-TB in Georgia are guided by the National Guidelines for the Management of TB. Results on drug susceptibility are obtained from the rapid tests such as Xpert MTB/RIF diagnostic technology, from molecular testing methods such as line probe assay as well as from conventional testing on solid and liquid media [13]. RR/MDR-TB patients receive second-line anti-TB drugs with at least five active drugs based on the individual resistance profile for a period of 18–20 months. In total, 28 drugs for the RR/MDR-TB patients were available in Georgia during the study period (Table 1). Patients with severe form of the disease are often hospitalized during the first two months of the treatment. We used the WHO definitions of TB treatment outcomes, as set out in Table 2.

### Adherence strategies

Each RR/MDR-TB patient received a patient-centered package of adherence interventions that included optional choice of DOT or VOT treatment supervision model, financial support, education and psychosocial support. The various adherence components were introduced in a phased approach between 2003 and 2017 (Table 3).

*DOT* was the first intervention introduced in 2003. Patients on DOT visit the healthcare facility on a daily basis to receive TB treatment drugs under direct observation of a nurse. If patients do not show up for a daily dose, adherence consultants call them the next day. If a patient misses at least three days of anti-TB medication consecutively, an adherence consultant informs the TB physician. Home-based DOT was available only for patients with special needs, such as the elderly and those with disability who were not able to come to the health facility. Home-based DOT was introduced together with the facility-based DOT.

The *VOT* treatment supervision model was implemented in 2016. Patients on VOT visit the TB facility weekly for anti-TB drug refills. VOT was provided by Skype, Viber or a TB application. Patients who have chosen Skype or Viber swallow their medications in front of a computer or smartphone camera and a nurse watches this action remotely and then documents the action in the treatment record. The TB application is an example of asynchronous VOT, when patients video-record their medication ingestion and nurses watch them later. Patients needed to have access to the mentioned technology to be eligible for VOT.

Since 2009, RR/MDR-TB patients have been receiving *adherence incentives* (“payment for performance”) and *transport cost reimbursement*. Adherence incentives (100 lari or ≈ 35 U.S. dollars) were provided on a monthly basis for patients who have administered 100% of their intended doses. Reimbursement of public transport costs was also provided weekly (money transfers to the patient’s bank account). Patients did not receive reimbursement for travel costs in advance.

*OAS* was introduced in June 2017. It is a package of education and psychosocial support offered to all RR/MDR-TB patients by specialized trained adherence consultants in the TB facilities (NCLTD or regional TB cabinets). Each patient received initial counseling by a TB physician who prescribes the treatment and then invites an adherence consultant to assess the patients’ needs, describe health system navigation and the treatment process and schedule follow-up visits. Education was delivered in group or individual sessions in a standardized manner, and each patient received at least two sessions during the course of the treatment – an initial session on the day of the diagnosis and another session at the end of the inpatient intensive phase treatment. These two sessions were mandatory. Additional sessions were provided based on patients’ needs. Psychosocial support and adherence counseling were provided as needed and on an individual basis. Patients with poor adherence and those returning after LTFU received additional counseling and support by adherence consultants. No information were available on duration and frequency of OAS educational sessions; % of patients in the post-OAS group who received both sessions; percentage of patients who received the different types of incentives and transport reimbursements pre and post OAS.

### Study population

The study population included all adult RR/MDR-TB patients who started treatment under DOT in the NCTLD in Tbilisi, Georgia during the period before (June 2015 – January 2016) and after (June 2017 – January 2018) OAS was introduced.

### Data sources and variables

Variables related to the study’s objectives were obtained from three different sources. Data related to demographic, behavioral and clinical characteristics of patients were obtained from the TB surveillance database. Variables related to treatment adherence such as days on anti-TB medication and maximum number of consecutive days without anti-TB medication were obtained form paper-based patient records and adherence monitoring forms filled by nurses in DOT units. Data from the TB surveillance database were exported into Microsoft Excel^®^ 2013. Data from paper-based information were manually entered into the excel file and records were matched using patient ID. To validate the data, 10% of the records were double-entered into two separate Excel files and discordances resolved by cross checking with the paper records. Logic checks were applied to ensure consistency of data.

### Statistical analysis

Data were then analyzed using R, version 3.5.2 software (© R Foundation for Statistical Computing, 2016). Socio-demographic and clinical characteristics of patients were summarized with proportions for categorical variables and means and standard deviation for numerical variables (age). Two-proportion Z-test and T-test were calculated to examine the difference in categorical and numerical patients’ characteristics respectively before and after the OAS. Adherence was calculated as a proportion of days covered by anti-TB medication during the whole treatment period. Denominator for the proportion was a time period between treatment outcome date and date of treatment start excluding Sundays (patients received treatment 6 days per week). We estimated the proportion of patients with adequate adherence defined as ≥ 85% of days covered by anti-TB medication. In addition, we estimated the proportion of patients with perfect adherence (100% of days covered by anti-TB medication). Among patients classified as adherent according to the programmatic definition before and after OAS, we calculated the median and interquartile ranges (IQR) of the maximum consecutive number of days without medication during the treatment period. Differences in medians were measured using Kruskal-Wallis test. Treatment outcomes were expressed in proportions. Absolute differences in treatment outcomes were compared using 95% confidence intervals (95%CI) for proportion difference and two-proportion Z-tests. Also we explored interactions between treatment success, cohort (Before / After) and other covariates using Poisson regressions with robust standard errors. Interactions allowed to assess whether the treatment success varied before and after the OAS within specific socio-demographic, clinical or behavioral subgroups. Levels of significance were set at 5%.

### Ethics

Permission to conduct the study was secured from the National Ethics Committee of the NCTLD and the Ethics Advisory Committee of the International Union against TB and Lung Disease, Paris, France.

## Results

### Socio-demographic and clinical characteristics

In total, we included 221 RR/MDR-TB patients in the study. Of whom, 114 (52%) and 107 (48%) patients initiated on TB treatment before and after introducing the OAS, respectively. There was no statistically significant difference in the socio-demographic and clinical characteristics of patients in the before and after cohorts (Table 4 A, B). In both cohorts, most patients (167, 76%) were male with a mean age of 41 ± 14 years.

Of all patients, 171 (77%) were unemployed; 34 (15%) had a history of incarceration, 17 (8%) had a history of alcohol abuse, and 5 (2%) patients had a history of drug abuse. Majority of the patients (200, 90%) had pulmonary TB and 92 (42%) patients were previously treated for TB. Of all the patients, 19 (9%) were people living with Human Immunodeficiency Virus (HIV), 52 (24%) had hepatitis C and 25 (11%) patients were diabetic.

### Adherence to treatment

**Figure 1.**
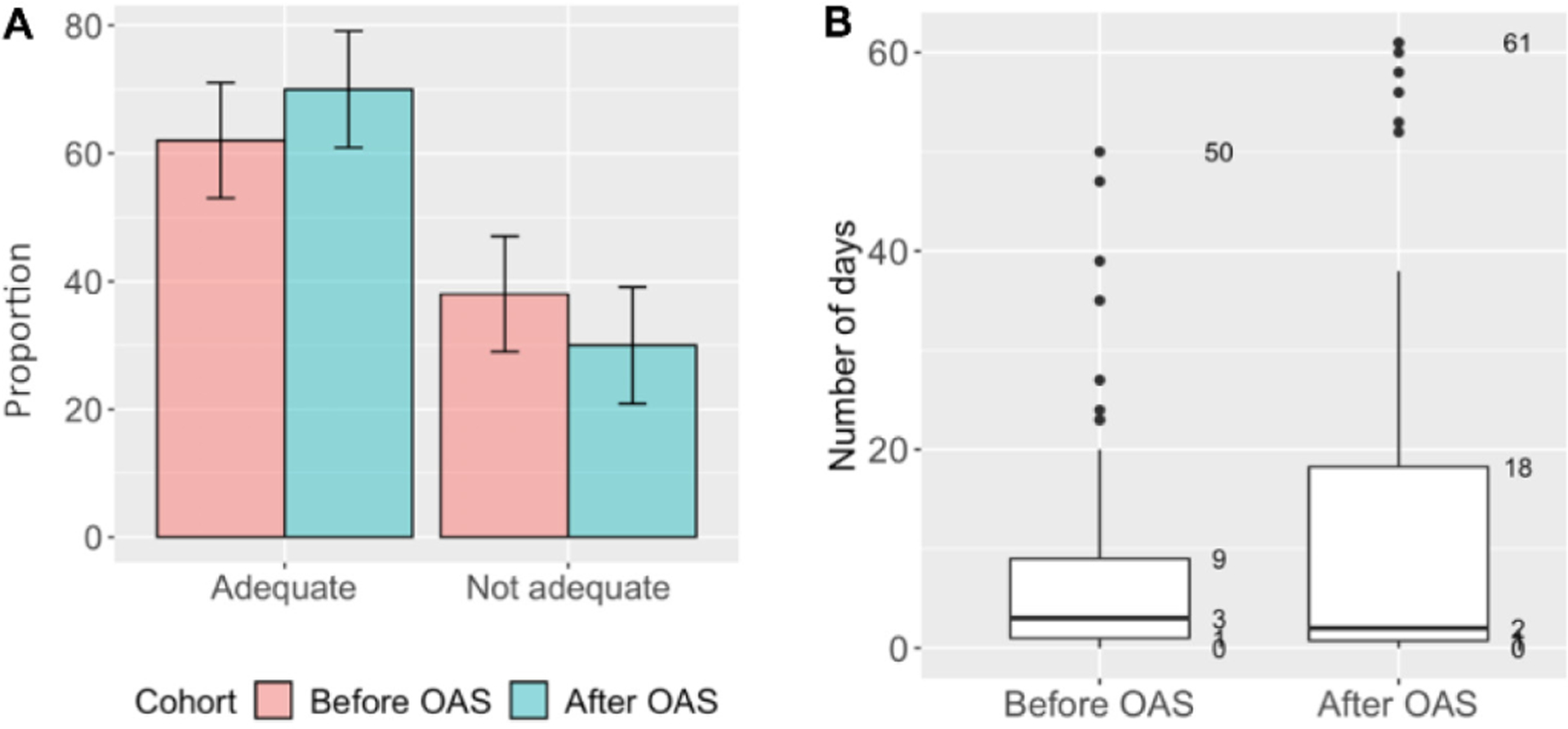
Treatment adherence among RR/MDR-TB adult patients enrolled for TB treatment before (June 2015 – January 2016) and after (June 2017 – January 2018) introducing OAS, Tbilisi, Georgia (N=208)*. **A.** Proportion of patients with adequate adherence** (Chi-square, p=0.290). **B.** Maximum of consecutive days without TB medication among patients with ≥85% of days covered by anti-TB medication (Kruskal-Wallis, p=0.588). Boxes represent 50% of the most frequent durations of interruptions in days. Bold horizontal line within the box is a median duration of interruptions. *Missing data (n=13) was excluded in the ‘Before’ (n=3) and ‘After’ (n=10) cohorts. **Adequate adherence was defined as ≥85% of days covered by anti-TB medication. *Abbreviations*: MDR: multidrug-resistant; OAS: optimized adherence support; RR: rifampicin-resistant; TB: tuberculosis.

Adherence data was available for 208 (94%) patients: 111/114 (97%) in the before and 97/107 (91%) in the after cohort. Smaller proportion of patients (62%, 69/111) had adequate adherence in the ‘before’ cohort compared to the ‘after’ cohort (70%, 68/97; *p* = 0.290) (Figure 1A). Of patients who achieved adequate adherence, 16/69 (23%) had perfect adherence in the ‘before’ cohort compared to 28/68 (41%) in the ‘after’ cohort (*p* = 0.038). Among patients with adequate adherence, the median maximum number of consecutive days without anti-TB medication was 3 days [IQR: 1–9] in the ‘before’ cohort and 2 days [IQR: 1–18] in the ‘after’ cohort (p=0.588, Figure 1B). However, 16% (11/69) and 32% (22/68) of the patients with adequate adherence in the ‘before’ and ‘after’ cohorts, respectively, had ≥ 2 consecutive weeks without TB medication (*p* = 0.041).

### Treatment outcomes

Treatment outcomes of the two cohorts are shown in Table 5. We did not find any significant differences in the treatment outcomes before and after the OAS. Overall treatment success was 64% and 63% in the ‘before’ and ‘after’ cohorts, respectively (*p* = 0.937). Treatment outcome was missing and recorded as ‘not evaluated’ in 3% (3/114) and 13% (13/107) of patients in the ‘before’ and ‘after’ cohorts, respectively (*p* = 0.014).

**Figure 2.**
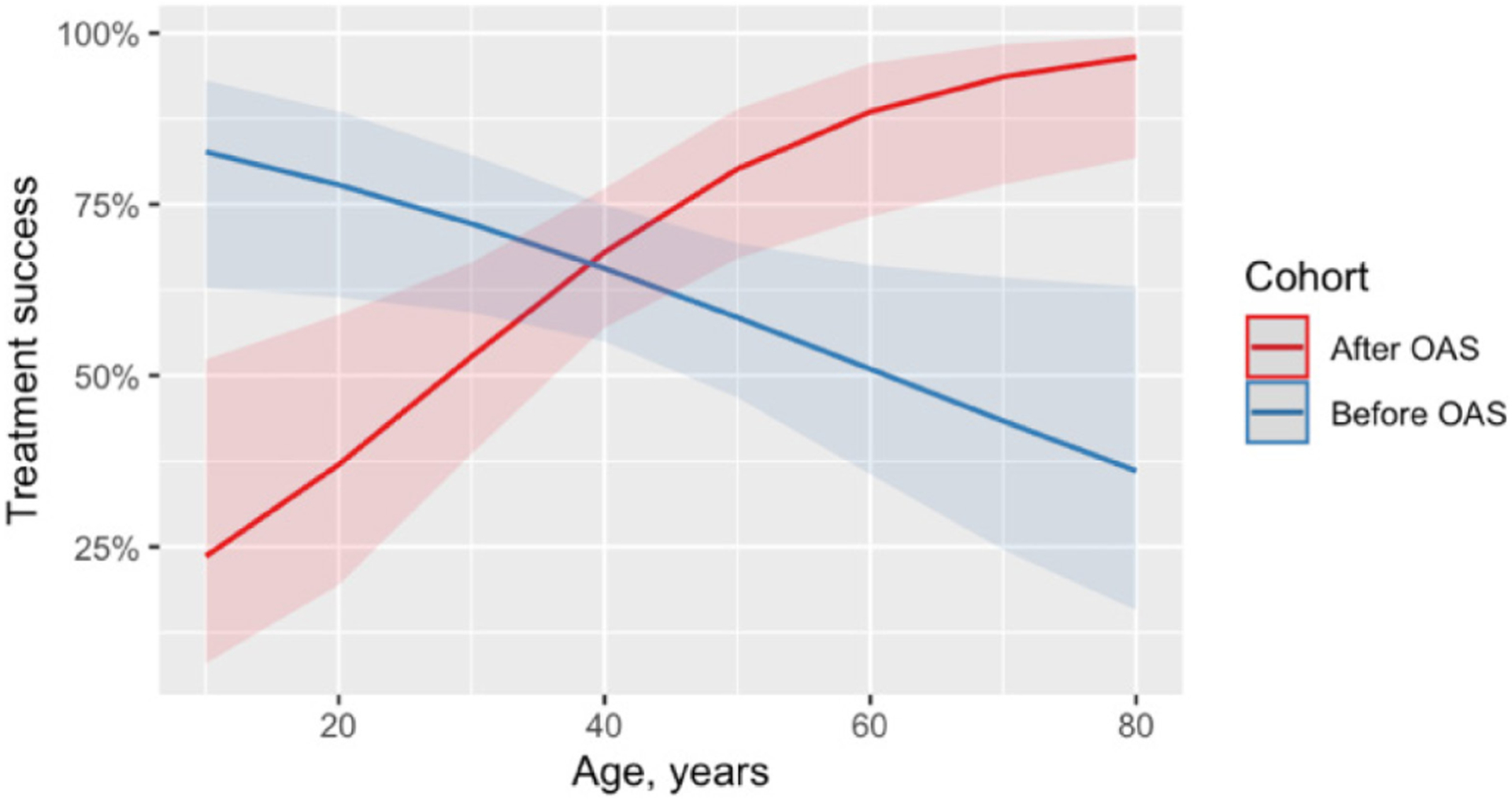
Association between age and treatment success among adult MDR/RR-TB patients enrolled for TB treatment before (June 2015 – January 2016) and after (June 2017 – January 2018) introducing OAS, Tbilisi, Georgia. Figure shows estimated trends for the association between age in years and proportion of patients with treatment success (cured or completed) before and after introducing OAS. The ribbon indicates 95% confidence interval. The trends were derived from adjusted Poisson regression with robust standard errors. Association between the age and treatment success was adjusted for alcohol abuse, history of imprisonment and human immunodeficiency virus. MDR: multidrug-resistant; OAS: optimized adherence support; RR: rifampicin-resistant; TB: tuberculosis.

In interaction analysis, we found that treatment success significantly increased with age in the ‘after’ cohort compared to an opposite trend in the ‘before’ cohort (Figure 2). Adjusted relative risk (aRR) of treatment success by age in years in the ‘before’ and ‘after’ cohorts was 1.02 (95%CI: 1.01–1.03, *p* = 0.006) and 0.99 (95% CI: 0.98–0.99, *p* = 0.042) respectively. No statistical differences in treatment success were found before and after OAS within other subgroups/all other variables.

## Discussion

This is the first study assessing the effect of introducing OAS (psychosocial support, education and adherence counseling) on treatment adherence and treatment outcomes among RR/MDR-TB patients in Georgia. The study found that introducing OAS has increased proportion of people with 85% or more adherence to treatment by 8%, but it had no significant impact on treatment outcomes. Treatment success rate remained far below the End TB strategy target of 90% [14]. An increase in treatment success rate was observed in elderly suggesting that this subgroup of patients may have benefited from the home-based DOT combined with OAS which is associated with lower LTFU than DOT at health facility [9].

While some studies found that provision of educational and psychosocial support to drug-resistant TB patients was associated with an increased adherence to treatment and subsequent improvement in treatment outcomes [12,15], other studies reported no effect on treatment outcomes despite the increase in adherence [9].

In our study, possible reasons for the lack of improvement in overall treatment outcomes can be explained by the fact that although seven in ten of the patients in the ‘After’ cohort achieved adequate adherence, one third of them interrupted treatment for ≥ 2 consecutive weeks compared to only one in six in the ‘Before’ cohort. Long treatment interruptions may reduce the concentration of drugs in the body or lead to development of additional resistance, therefore, increasing the likelihood of treatment failure [16]. Another explanation is that the obtained difference in the adherence between the cohorts was simply not big enough to have an impact on treatment outcomes [17]. Other factors such as drug toxicity and intolerability may decrease adherence to treatment, increase LTFU and lead to poor treatment outcomes [18]. However, the treatment success rate was 64% prior to introduction of OAS and 63% thereafter. These treatment success levels are higher than the global average reported by WHO and stands at 56% [5]. Previous systematic reviews that have assessed treatment success using the 24-month regimen in 23 countries have shown an average treatment success rate of 54% [19]. This implies that even before the introduction of OAS, the NCTLD in Tbilisi was already achieving treatment success level higher than the global average with a 24-month regimen. Considering also that an adherence package existing prior to the OAS, any additive benefits remain limited and therefore difficult to demonstrate. This is all the more logical since the financial incentives were already being offered prior to introduction of OAS and thus the only added intervention in the OAS group was reinforcement of counseling and education intervention. To further improve adherence and increase treatment success, there is a need for shorter duration regimens that are less toxic, less expensive, with minimum adverse effects. To this effect, the NCTLD in Georgia is currently assessing the effectiveness and safety of all-oral modified shorter MDR-TB treatment regimen.

The strengths of the study are: i) the data were obtained from a routine programme setting and therefore are likely to reflect operational reality on the ground; ii) data encoders were well trained and supervised, and we therefore believe the data were robust; and iii) we adhered to the STROBE (Strengthening the Reporting of Observational Studies in Epidemiology) guidelines for reporting on observational studies [20]. Limitations are: i) high proportions with unknown outcome in the ‘after’ cohort; ii) we were unable to assess the fidelity of implementation of OAS retrospectively; iii) we were unable to investigate the reasons for LTFU which is beyond the scope of this study; iv) adherence data was missing for 6% of patients which could’ve impacted measurements and v) our study site included only a capital region while OAS has been already scaled up to the whole country. Another limitation was the large difference in the proportion of missing data (0% vs 65%) on diabetes mellitus present in two groups and it was not clear why there was such difference as we did not have control over the initial collection of the data.

Despite the limitations our study showed a number of operational shortcomings that merits further discussion. First, adherence incentive was only offered to those who achieved 100% adherence in both cohorts. This demotivate those who achieved the program target for adherence which is set at 85% and may negatively influence adherence and lead to missed visits and eventually LTFU. The increase in the percentage of patients with treatment interruption of ≥ 2 consecutive weeks after the introduction of OAS (among patients with adequate adherence) could be a direct result of the disincentivize policy of those who achieved the programme target for adherence. Second, patients receive reimbursement of transport costs on a weekly basis. However, seven in 10 patients were not employed and may not have money to cover the transport cost in the first place. A way forward could be to assess the financial capability of each patient individually and provide financial support in advance for those in need. Third, additional psychosocial support and counseling sessions were offered according to the needs expressed by patients. There was no set provision of psychosocial and counseling support for specific vulnerable populations. Our cohorts included subgroups of patients who were elderly, unemployed, have comorbidities, had a history of incarceration, consumed alcohol and used drugs. Such patients should be prioritized for intensive follow-up care and additional adherence support, as they are vulnerable and at high risk of LTFU [21]. Finally, there was no system for recording the coverage of OAS components per patient. The number of adherence support sessions received could have an impact on adherence and treatment outcomes [22].

## Conclusions

We found that implementation of OAS package, which includes psychosocial support, education and adherence case-management in addition to treatment supervision and financial support had limited effect on adherence and did not improve treatment outcomes among RR/MDR-TB patients.

## Figures and Tables

**Table 1.** Second-line antituberculosis drugs available in the treatment regimens for rifampicin- and multidrug- resistant tuberculosis patients during the study period (2015–2018).

**Group 1.**	First line oral antituberculosis drugs	Pirazinamide, Ethambutol, Rifabuton
**Group 2.**	Injectable antituberculosis drugs	Kanamycin, Amikacin, Capreomycin, Streptomycin
**Group 3.**	Fluoroquinolones	Levofloxacin, Moxifloxacin, Gatifloxacin
**Group 4.**	Oral, bacteriostatic second-line antituberculosis drugs	Ethionamide, Prothionamide, Cycloserine, Terisidone, Paraaminosalicylic acid (PAS), PAS+Na,
**Group 5.**	Antituberculosis drugs with limited data for efficacy and/or long-term safety in the treatment of drug-resistant tuberculosis	Bedaquiline, Delamanid, Linezolid, Clofazimine, Amoxacillin, Clavulanate, Imipenem, Cilastatin, Meropenem, High-dose isoniazid, Thioacetazon, Clarithromycin

**Table 2.** World Health Organization definitions of tuberculosis treatment outcomes.

**Cured:**	Treatment completed without evidence of failure, and three or more consecutive cultures taken at least 30 days apart are negative at the end of treatment.
**Treatment completed:**	Treatment completed without evidence of failure but no record that three or more consecutive cultures taken at least 30 days apart are negative at the end of treatment.
**Treatment failed:**	Treatment terminated or need for permanent regimen change of ≥ 2 antituberculosis drugs because of: a) lack of conversion, b) bacteriological reversion after conversion to negative, c) evidence of acquired resistance to drugs in the shorter regimen or adverse drug reactions leading to the change of at least two antituberculosis drugs in the regimen.
**Died:**	A patient who dies for any reason during the course of treatment.
**Lost to follow-up:**	A patient whose treatment was interrupted for 2 consecutive months or more.
**Not evaluated:**	A patient with an unknown treatment outcome including patients that were transferred out.

**Table 3.** Timeline of adherence interventions for rifampicin- and multidrug- resistant tuberculosis patients in Georgia.

Characteristics		2003 – 2008	2009 – 2015	2016 – May 2017	June 2017 – present
**Treatment supervision**	Directly observed therapy	+	+	+	+
	Video-observed therapy			+	+
**Financial support**	Adherence incentives		+	+	+
	Transport cost reimbursement		+	+	+
**Optimized adherence support**	Educational sessions				+
	Psychosocial and adherence support				+

**Table 4a.** Demographic and behavioral characteristics. Sociodemographic, behavioral and clinical characteristics of adult RR/MDR-TB patients enrolled for TB treatment before (June 2015 – January 2016) and after (June 2017 – January 2018) introducing OAS, Tbilisi, Georgia (N = 221).

Characteristics	Total (N = 221)*	Before OAS (N = 114)*	After OAS (N = 107)*	p-value**
**Age, years (mean±SD)**	41 ± 14	41 ± 15	41 ± 13	0.681
**Age groups**				
18–34	81 (37%)	44 (39%)	37 (35%)	0.632
35–44	48 (22%)	19 (17%)	29 (27%)	0.086
45–59	67 (30%)	37 (32%)	30 (28%)	0.570
≥60	25 (11%)	14 (12%)	11 (10%)	0.797
**Gender**				
Male	167 (76%)	84 (74%)	83 (78%)	0.606
Female	54 (24%)	30 (26%)	24 (22%)	-
**History of imprisonment**				
Yes	34 (15%)	16 (14%)	18 (17%)	0.699
No	178 (81%)	94 (82%)	84 (79%)	0.568
Not recorded	9 (4%)	4 (4%)	5 (5%)	0.923
**Employment**				
Employed	37 (17%)	18 (16%)	19 (18%)	0.833
Unemployed	171 (77%)	92 (81%)	79 (74%)	0.290
Not recorded	13 (6%)	4 (4%)	9 (8%)	0.207
**Alcohol abuse**				
Yes	17 (8%)	13 (11%)	4 (4%)	0.059
No	201 (91%)	99 (87%)	102 (95%)	0.050
Not recorded	3 (1%)	2 (2%)	1 (1%)	1.000
**Drug abuse**				
Yes	5 (2%)	2 (2%)	3 (3%)	0.943
No	168 (76%)	90 (79%)	78 (73%)	0.371
Not recorded	48 (22%)	22 (19%)	26 (24%)	0.461

* Data is summarized with frequencies and percentages unless otherwise stated.

** T-test for comparison of means and two proportions Z-test for categorical variables.

HIV: human immunodeficiency virus; MDR: multi-drug-resistant; OAS: optimized adherence support; RR: rifampicin-resistant; SD: standard deviation; TB: tuberculosis.

**Table 4b.** Clinical characteristics. Sociodemographic, behavioral and clinical characteristics of adult RR/MDR-TB patients enrolled for TB treatment before (June 2015 – January 2016) and after (June 2017 – January 2018) introducing OAS, Tbilisi, Georgia (N = 221).

Characteristics	Total (N = 221)*	Before OAS (N = 114)*	After OAS (N = 107)*	p-value**
**Type of resistance**				
RR-TB	25 (11%)	11 (10%)	14 (13%)	0.553
MDR-TB	177 (80%)	92 (81%)	85 (79%)	0.947
Not recorded	19 (9%)	11 (10%)	8 (7%)	0.737
**TB location**				
Pulmonary	200 (90%)	104 (91%)	96 (90%)	0.879
Extra-pulmonary	21 (10%)	10 (9%)	11 (10%)	–
**History of TB treatment**				
New	129 (58%)	67 (59%)	62 (58%)	1.000
Previously treated	92 (42%)	47 (41%)	45 (42%)	–
**HIV status**				
Positive	19 (9%)	8 (7%)	11 (10%)	0.532
Negative	194 (88%)	100 (88%)	94 (88%)	1.000
Not recorded	8 (4%)	6 (5%)	2 (2%)	0.322
**Hepatitis C status**				
Positive	52 (24%)	25 (22%)	27 (25%)	0.675
Negative	82 (37%)	45 (39%)	37 (35%)	0.540
Not recorded	87 (39%)	44 (39%)	43 (40%)	0.917
**Diabetes Mellitus**				
Yes	25 (11%)	13 (11%)	12 (12%)	0.999
No	126 (57%)	101 (89%)	25 (23%)	<0.001
Not recorded	70 (32%)	0 (0%)	70 (65%)	<0.001

* Data is summarized with frequencies and percentages unless otherwise stated.

** T-test for comparison of means and two proportions Z-test for categorical variables.

HIV: human immunodeficiency virus; MDR: multi-drug-resistant; OAS: optimized adherence support; RR: rifampicin-resistant; SD: standard deviation; TB: tuberculosis

**Table 5.** Final treatment outcomes among adult RR/MDR-TB patients enrolled for TB treatment before (June 2015 – January 2016) and after (June 2017 – January 2018) introducing OAS, Tbilisi, Georgia (N = 221)

Final treatment outcomes	Total (N = 221)		Before OAS (N = 114)		After OAS (N = 107)		Percent difference	P-value*
	N	(%)	N	(%)	N	(%)	[95% CI]	
Treatment success**	140	(63)	73	(64)	67	(63)	−1 [−14, 11]	0.937
Cured	116	(52)	60	(53)	56	(52)	−1 [−13, 14]	1.000
Completed	24	(11)	13	(11)	11	(10)	−1 [−8, 10]	0.959
Failure	7	(3)	6	(5)	1	(1)	−4 [−1, 10]	0.147
Lost to follow up	49	(22)	27	(24)	22	(21)	−3 [−9, 15]	0.692
Died	9	(4)	5	(4)	4	(4)	0 [−4, 4]	1.000
Not evaluated	16	(7)	3	(3)	13	(12)	9 [3,16]	**0.014**

* Two-proportion Z-test.

** Treatment success is a sum of ‘cured’ and ‘completed’.

CI: confidence interval; MDR: multidrug-resistant; OAS: optimized adherence support; RR rifampicin-resistant; TB: tuberculosis.

## References

[1] Dheda K, Gumbo T, Maartens G, Dooley KE, McNerney R, Murray M, Furin J, Nardell EA, London L, Lessem E, Theron G, van Helden P, Niemann S, Merker M, Dowdy D, Van Rie A, Siu GK, Pasipanodya JG, Rodrigues C, Clark TG, Sirgel FA, Esmail A, Lin HH, Atre SR, Schaaf HS, Chang KC, Lange C, Nahid P, Udwadia ZF, Horsburgh CR Jr, Churchyard GJ, Menzies D, Hesseling AC, Nuermberger E, McIlleron H, Fennelly KP, Goemaere E, Jaramillo E, Low M, Jara CM, Padayatchi N, Warren RM (2017) The epidemiology, pathogenesis, transmission, diagnosis, and management of multidrug-resistant, extensively drug-resistant, and incurable tuberculosis. Lancet Respir Med S2213–2600: 30079–6. 10.1016/S2213-2600(17)30079-6 28344011

[2] Koch A, Cox H, Mizrahi V (2018) Drug-resistant tuberculosis: challenges and opportunities for diagnosis and treatment. Curr Opin Pharmacol 42: 7–15. 29885623 10.1016/j.coph.2018.05.013 PMC6219890

[3] Thomas BE, Shanmugam P, Malaisamy M, Ovung S, Suresh C, Subbaraman R, Adinarayanan S, Nagarajan K (2016) Psycho-socio-economic issues challenging multidrug resistant tuberculosis patients: a systematic review. PLoS One 11: e0147397. 26807933 10.1371/journal.pone.0147397 PMC4726571

[4] Law S, Daftary A, O’Donnell M, Padayatchi N, Calzavara L, Menzies D (2019) Interventions to improve retention-in-care and treatment adherence among patients with drug-resistant tuberculosis: A systematic review. Eur Respir J. 53: 1801030. 30309972 10.1183/13993003.01030-2018

[5] World Health Organization (2019) Global Tuberculosis Report 2019. Geneva, Switzerland. Available: https://apps.who.int/iris/bitstream/handle/10665/329368/9789241565714-eng.pdf. Accessed 27 August 2020.

[6] Munro SA, Lewin SA, Smith HJ, Engel ME, Fretheim A, Volmink J (2007) Patient adherence to tuberculosis treatment: A systematic review of qualitative research. PLoS Med 4: e238. 17676945 10.1371/journal.pmed.0040238 PMC1925126

[7] Müller AM, Osório CS, Silva DR, Sbruzzi G, De Tarso P, Dalcin R (2018) Interventions to improve adherence to tuberculosis treatment: Systematic review and meta-analysis. Int J Tuberc Lung Dis 22: 731–740. 29914598 10.5588/ijtld.17.0596

[8] Sharma SK, Dheda K (2019) What is new in the WHO consolidated guidelines on drug-resistant tuberculosis treatment? Indian J Med Res 149: 309–312. 31249191 10.4103/ijmr.IJMR_579_19 PMC6607808

[9] Alipanah N, Jarlsberg L, Miller C, Linh NN, Falzon D, Jaramillo E, Nahid P (2018) Adherence interventions and outcomes of tuberculosis treatment: A systematic review and meta-analysis of trials and observational studies. PLoS Med 15: e1002595. 29969463 10.1371/journal.pmed.1002595 PMC6029765

[10] Ngwatu BK, Nsengiyumva NP, Oxlade O, Mappin-Kasirer B, Nguyen NL, Jaramillo E, Falzon D, Schwartzman K; Collaborative group on the impact of digital technologies on TB (2018) The impact of digital health technologies on tuberculosis treatment: A systematic review. Eur Respir J 51: 1701596. 29326332 10.1183/13993003.01596-2017 PMC5764088

[11] Deshmukh RD, Dhande DJ, Sachdeva KS, Sreenivas AN, Kumar AMV, Parmar M (2018) Social support a key factor for adherence to multidrug-resistant tuberculosis treatment. Indian J Tuberc 65: 41–47. 29332647 10.1016/j.ijtb.2017.05.003

[12] Tola HH, Shojaeizadeh D, Tol A, Garmaroudi G, Yekaninejad MS, Kebede A, Ejeta LT, Kassa D, Klinkenberg E (2016) Psychological and educational intervention to improve tuberculosis treatment adherence in Ethiopia based on health belief model: A cluster randomized control trial. PLoS One 11: e0155147. 27167378 10.1371/journal.pone.0155147 PMC4864292

[13] Ministry of health in Georgia (2019) [National Strategy for Tuberculosis Control 2019–2022]. Available: http://www.georgia-ccm.ge/wp-content/uploads/National-Strategy-for-Tuberculosis-Control-in-Georgia-2019-2022.pdf. Accessed 27 August 2020.

[14] World Health Organization (2015) The End TB Strategy. Geneva, Switzerland. Available: http://www.who.int/tb/post2015_TBstrategy.pdf. Accessed 27 August 2020.

[15] Pradipta IS, Houtsma D, van Boven JFM, Alffenaar JWC, Hak E (2020) Interventions to improve medication adherence in tuberculosis patients: a systematic review of randomized controlled studies. NPJ Prim Care Respir Med 30: 21. 32393736 10.1038/s41533-020-0179-x PMC7214451

[16] Tola HH, Holakouie-Naieni K, Mansournia MA, Yaseri M, Tesfaye E, Mahamed Z, Sisay MM (2019) Intermittent treatment interruption and its effect on multidrug resistant tuberculosis treatment outcome in Ethiopia. Sci Rep 9: 20030. 31882784 10.1038/s41598-019-56553-1 PMC6934462

[17] Tesfahuneygn G, Medhin G, Legesse M (2015) Adherence to anti-tuberculosis treatment and treatment outcomes among tuberculosis patients in Alamata District, northeast Ethiopia. BMC Res Notes 8: 503. 26420164 10.1186/s13104-015-1452-x PMC4588463

[18] Dela AI, Tank NKD, Singh AP, Piparva KG (2017) Adverse drug reactions and treatment outcome analysis of DOTS-plus therapy of MDR-TB patients at district tuberculosis centre: A four year retrospective study. Lung India 34: 522–526. 29098997 10.4103/0970-2113.217569 PMC5684809

[19] Ahuja SD, Ashkin D, Avendano M, Banerjee R, Bauer M, Bayona JN, Becerra MC, Benedetti A, Burgos M, Centis R, Chan ED, Chiang CY, Cox H, D’Ambrosio L, DeRiemer K, Dung NH, Enarson D, Falzon D, Flanagan K, Flood J, Garcia-Garcia ML, Gandhi N, Granich RM, Hollm-Delgado MG, Holtz TH, Iseman MD, Jarlsberg LG, Keshavjee S, Kim HR, Koh WJ, Lancaster J, Lange C, de Lange WC, Leimane V, Leung CC, Li J, Menzies D, Migliori GB, Mishustin SP, Mitnick CD, Narita M, O’Riordan P, Pai M, Palmero D, Park SK, Pasvol G, Peña J, Pérez-Guzmán C, Quelapio MI, Ponce-de-Leon A, Riekstina V, Robert J, Royce S, Schaaf HS, Seung KJ, Shah L, Shim TS, Shin SS, Shiraishi Y, Sifuentes-Osornio J, Sotgiu G, Strand MJ, Tabarsi P, Tupasi TE, van Altena R, Van der Walt M, Van der Werf TS, Vargas MH, Viiklepp P, Westenhouse J, Yew WW, Yim JJ; Collaborative Group for Meta-Analysis of Individual Patient Data in MDR-TB (2012) Multidrug resistant pulmonary tuberculosis treatment regimens and patient outcomes: an individual patient data meta-analysis of 9,153 patients. PLoS Med 9: e1001300. 22952439 10.1371/journal.pmed.1001300 PMC3429397

[20] von Elm E, Altman DG, Egger M, Pocock SJ, Gøtzsche PC, Vandenbroucke JP (2014) The strengthening the reporting of observational studies in epidemiology (STROBE) statement: Guidelines for reporting observational studies. Int J Surg 12: 1495–1499.25046131

[21] Kuchukhidze G, Kumar AMV, De Colombani P, Khogali M, Nanava U, Blumberg HM, Kempker RR (2014) Risk factors associated with loss to follow-up among multidrug-resistant tuberculosis patients in Georgia. Public Health Action 4: S41–S46. 26393097 10.5588/pha.14.0048 PMC4547510

[22] Voils CI, King HA, Maciejewski ML, Allen KD, Yancy WS, Shaffer JA (2014) Approaches for informing optimal dose of behavioral interventions. Ann Behav Med. 48: 392–401. 24722964 10.1007/s12160-014-9618-7 PMC4414086

